# Colonization of Extramammary Sites with Mastitis-Associated *S. aureus* Strains in Dairy Goats

**DOI:** 10.3390/pathogens12040515

**Published:** 2023-03-26

**Authors:** Catharina Elizabeth Exel, Yvette de Geus, Mirlin Spaninks, Gerrit Koop, Lindert Benedictus

**Affiliations:** 1 Department Population Health Sciences, Division Farm Animal Health, Utrecht University, Yalelaan, 7, 3584 CL Utrecht, The Netherlands; 2 Department Population Health Sciences, Division Institute for Risk Assessment Sciences, Utrecht University, Yalelaan, 7, 3584 CL Utrecht, The Netherlands

**Keywords:** *Staphylococcus aureus*, dairy goats, intramammary infection, extramammary sites, colonization

## Abstract

*Staphylococcus aureus* (*S. aureus*), a major mastitis pathogen in dairy goats, is classified as a contagious pathogen. Although previous research has shown that extramammary body sites can be colonized with *S. aureus*, it is unknown whether these sites are reservoirs for intramammary infections. The aim of this research was to determine whether extramammary sites can be colonized with mastitis-associated *S. aureus* strains in dairy goats. Milk samples were collected from 207 primiparous goats and from 120 of these goats, extramammary site samples (hock, groin, nares, vulva and udder) were collected from a large commercial dairy goat herd in the Netherlands during four sampling visits. Extramammary site swabs and milk samples were (selectively) cultured and *S. aureus* isolates were *spa* genotyped. The prevalence of colonization of the extramammary sites at goat level was 51.7% and the prevalence of *S. aureus* intramammary infections was 7.2%. The nares were colonized most frequently (45%), while the groin area was colonized the least (2.5%). Six *spa* genotypes were identified in this herd and there was no significant difference in the distribution of *spa* genotypes between the milk or the extramammary sites (*p* = 0.141). Both in the extramammary sites and in the milk, *spa* genotypes t544 (82.3% and 53.3%) and t1236 (22.6% and 33.3%) were the dominant genotypes. These results show that in goats, extramammary sites, particularly the nares, are frequently colonized with mastitis-associated *S. aureus* strains. Extramammary sites may, thus, be a source of *S. aureus* intramammary infections that are not targeted by the intervention measures aimed at preventing transmission from infected udder glands.

## 1. Introduction

*Staphylococcus aureus* (*S. aureus*) is a major cause of intramammary infections (IMI) in dairy goats worldwide and can lead to severe mastitis [1]. *Staphylococcus aureus* mastitis has detrimental effects on the health and wellbeing of goats, as well as an economic impact and is the main reason for the culling of dairy goats [2]. *Staphylococcus aureus* is classified as a contagious pathogen, with infected udder glands assumed to be the main reservoir of infection and transmission, mainly occurring during milking [1]. Control measures aimed at preventing contagious transmission are not always effective in eradicating *S. aureus* IMI from dairy cattle herds [3]. In cattle, there is evidence that extramammary site colonization could be a reservoir for IMI [3,4,5,6,7,8,9,10,11]. Additionally, in cows different *S. aureus* genotypes were shown to be associated with colonization of either extramammary sites, milk (IMI) or both sites [10,11]. In goats, several extramammary sites can be colonized by *S. aureus*, including teat skin, the vulva and the nares [12,13,14,15,16]. However, it is unknown whether *S. aureus* in extramammary sites may serve as reservoirs for *S. aureus* IMI in dairy goats, which would undermine the effectiveness of control measures aimed at preventing transmission from infected udder glands. The aim of this study was to determine to what extent extramammary sites of dairy goats are colonized with mastitis-associated *S. aureus* strains.

## 2. Materials and Methods

### 2.1. Herd and Animal Selection

The study was conducted on a commercial dairy goat herd in the Overijssel province in the Netherlands. A herd of 3000 dairy goats, mainly from the Saanen breed, were fed a mix of silage with concentrates, and were housed in a deep litter stable. The goats were milked twice per day, in an outside rotary milking parlor with 85 stands, and the average yearly milk yield was 1050 kg per goat. The farm was visited four times between April 2022 and June 2022. From primiparous goats’ milk, 207 samples were collected four times during consecutive visits, while 120 goat extramammary sites were sampled once (30 goats per visit). During each sampling visit, milk samples from both udder halves were collected from all primiparous goats with an uneven ear tag ID. For the extramammary sites, at each visit 30 random primiparous goats with a specific last number on their ear tag ID (i.e., 1, 3, 5 and 7, respectively) were sampled to avoid repeated sampling of the same animal. 

### 2.2. Sampling

The samples were collected and processed as previously described [10]. In short, the composite milk samples were collected aseptically from lactating goats. Ten microliters of milk were inoculated on Columbia agar plates supplemented with 5% sheep blood (BD Biosciences, Franklin Lakes, NJ USA) and incubated for 22–24 h at 37 °C. The following extramammary sites were swabbed using a sterile viscose swab (Sarstedt, Nümbrecht, Germany): hock skin (one leg, cleanest hock, hocks were checked for lesions and only hocks without lesions were sampled), groin (the swab was inserted between the hindleg and udder), udder skin (skin of the cleft between the left and right half of the udder), nares (the first 2 to 3 cm of the nasal cavity, rubbed circumferentially) and vulva (the labia were parted and the mucosa was gently swabbed). The extramammary sites were chosen based on previous research on goats and cattle that showed that these sites can be colonized with *S. aureus* [5,8,10,11,12,13,14,15]. For the sampling of the udder skin, the cleft of the udder was sampled, as it is distant from the teats and less likely to be contaminated by *S. aureus* from milk. The eluates of the swabs were cultured using a selective two-step high salt Mueller–Hinton broth and samples were, subsequently, plated as for the milk samples. 

### 2.3. Identification of Staphylococcus aureus

The identification of *S. aureus* from milk was performed using a matrix-assisted laser desorption/ionization time-of-flight analyzer (MALDI-TOF), when milk was identified as positive for *S. aureus* further typing of the isolate was performed as for the extramammary sites. The identification and typing of *S. aureus* colonies was conducted by colony PCR for the *S. aureus* specific *femA* [17] and *spa* [18] genes. The *spa* gene of all the *spa* PCR-positive isolates was sequenced and *spa* typing was performed (https://www.spaserver.ridom.de/; accessed on 15 September 2022). 

### 2.4. Data Analysis

The prevalence of the colonization of the extramammary sites was calculated by dividing the number of *S. aureus* positive swabs by the total number of swabs taken. Milk samples were taken four times from each goat and to account for the repeated sampling of animals, the period prevalence was used for milk (IMI). The period prevalence gives the proportion of goats testing positive for *S. aureus* at least once across the four samplings. The 95% confidence interval of the prevalence was calculated according to the Wilson method (https://epitools.ausvet.com.au/ciproportion; accessed on 19 January 2023). A new *S. aureus* IMI case was defined as the first milk sampling a goat tested positive for *S. aureus* in milk. To test whether *S. aureus spa* genotypes were associated with milk (IMI) or extramammary site colonization, a Fisher–Freeman–Halton exact test (n x m table) was performed using IBM SPSS Statistics [19] (Version 27, IBM, New York, NY USA). The *p*-values ≤0.05 were considered significant. The graphical abstract was created using BioRender.com.

## 3. Results and Discussion

Table 1 gives an overview of the number of milk samples and extramammary sites yielding positive culture of *S. aureus* and the prevalence of IMI or colonization. The prevalence of *S. aureus* IMI was 7.2% and the number of new *S. aureus* IMI varied between 1 and 9 cases for each sampling. Of the 120 goats sampled, 62 goats were colonized in at least one extramammary site (goat level colonization prevalence 51.7%), only ten goats were colonized in two extramammary sites and three goats were colonized in three extramammary sites (Table 1, Supplementary Table S1). The prevalence of colonization varied per extramammary site, with the nares having the highest prevalence of colonization at 45% and the groin the lowest at 2.5%. 

In line with this study, previous research on goats found colonization prevalence of the nares varied from 25%–68.9% [7,13,15,16]. The differences in prevalence could be caused by the sampling and culturing method, the country, the goat breed, the phenotypic characteristics of the *S. aureus* strains (e.g., the ability to transmit or colonize a site) and the season in which the samples were collected [13,14,15,16]. Previously, Mørk et al. [14] found the colonization prevalence of the nares to be higher before drying off (75.6%) than after kidding (62%), but that of the vulva to be lower before (19.2%) rather than after kidding (44.9%). This is evidence that colonization is dynamic and that the moment of sampling can influence the prevalence of colonization. Interestingly, all these studies found colonization prevalence of the nares in goats that were higher than those reported for dairy cattle [4,5,6,7,8,10]. The differences in colonization prevalence of the nares between cows and goats could be caused by housing and climate conditions, particularly dust concentration. On pig farms it has been shown that methicillin resistant *S. aureus* (MRSA) was present both in the dust and in the air and that this could be a potential source of the MRSA colonization of the nares both in humans and pigs [20,21,22,23]. Dust can also alter the nasal microbiome [24,25]. To our knowledge, nothing is known about the role of dust on *S. aureus* transmission in dairy goats. For future studies in dairy goats, we recommend sampling dust and air to determine the presence and load of *S. aureus*, to explore whether dust could play a direct role in colonization of the nares with *S. aureus* and potentially also in causing IMI.

An overview of the different *spa* genotypes found within *S. aureus* isolated from this herd and their distribution between the extramammary sites and milk is presented in Table 2. In this herd, *spa* genotype t544 (82.3%) and t1236 (22.6%) were the most dominant. Four other *spa* genotypes isolated were only found in one to three sites. Two isolates had a novel *spa* genotype and one *S. aureus* isolate was negative in the *spa* PCR and, therefore, non-typeable, as previously described [10,26,27]. In the nares of three goats, two *spa* genotypes were isolated simultaneously. The *spa* genotype combination in the nares was t544 with either t1236, t3992 or a novel *spa* genotype (Supplementary Table S1). Of all the goats colonized, three also had an *S. aureus* IMI during the sampling period. In all three cases, both in the milk and in the extramammary sites, the *spa* genotype t544 was isolated (Supplementary Table S1). Overall, we found no association between the site (milk or extramammary site) and the *S. aureus spa* genotype (*p* = 0.141, Fisher–Freeman–Halton exact test). The dominant *spa* genotypes, t544 and t1236, were found in similar proportions in the milk and extramammary sites and one of the three other genotypes found in milk were also identified in the extramammary sites. This showed that *S. aureus* strains with these two *spa* genotypes were able to both colonize extramammary sites and cause intramammary infections in dairy goats and, therefore, that extramammary sites were frequently colonized with mastitis-associated *S. aureus* strains. 

One of the dominant *S. aureus spa* genotypes found in this herd, t544, is frequently identified in IMI in goats and belongs to the small ruminant-associated clonal complex (CC) 133 [28,29,30,31,32]. Hoekstra et al. [28] showed that goat IMI strains with *spa* genotype t544 have a high production of the leukotoxin LukMF’, an important virulence factor for ruminant mastitis. Limited data is available for *spa* genotype t1236 in goats, but it has previously been observed in an IMI case in goats [30]. Several studies identified t1236 in IMI cases in cattle [33,34,35,36,37]. The t1236 *spa* genotype is associated with CC97, frequently found in mastitis in cattle and harbors a high frequency of methicillin-resistant strains [38,39,40,41] In goat, sheep and deer, strains from CC97 have been isolated from the nares [30,42,43,44,45,46].

Pulsed-field gel electrophoresis typing of *S. aureus* strains isolated from the nares, vulva and milk by Mørk et al. [14] indicated that similar strains could colonize milk and extramammary sites in goats. We showed that the *spa* genotypes of *S. aureus* isolates were found in similar proportions in milk and in extramammary sites. In dairy cattle it has been shown that specific *spa* genotypes can either be associated with colonization of extramammary sites, milk or both [10,11]. This is the first study using sequence-based genotyping to show that extramammary sites can be colonized with mastitis-associated *S. aureus* strains, indicating that extramammary sites are a potential reservoir for *S. aureus* IMI in dairy goats. Although this has never been studied, there could be spill-over from *S. aureus* of extramammary sites to the udder (within a goat or to another goat), by for example licking, self-sucking, rubbing or by other close physical contact. Even if the spill-over of extramammary *S. aureus* to milk is rare, subsequent udder-to-udder transmission (contagious transmission) could lead to a big impact on udder health. In a bioeconomic simulation model of a dairy cattle herd [47], we showed that spill-over *S. aureus* IMI influences the economic and epidemiologic outcome of control measures. Therefore, extramammary reservoirs should be taken into account in *S. aureus* control programs for dairy goats. *Staphylococcus aureus* genotypes can vary within and between goat herds [14,30,31,32] and phenotypic characteristics between *S. aureus* strains with a different *spa* genotype can vary [34,48,49]. The presence of *S. aureus* strains with different epidemiological and phenotypical characteristics within a herd could further complicate *S. aureus* control programs. The current study only sampled primiparous goats from a single herd and further studies are needed to characterize the variation between herds, between different parities and the differences between strains in association with milk and extramammary sites. 

Intramammary infections with *S. aureus* can occur in primiparous goats before parturition and, therefore, before they are lactating [50]. Dairy calves can already have an adaptive immune response against *S. aureus* at 12 weeks of age [51]. This indicates (transient) colonization of calves at a very young age. We hypothesize that the colonization of extramammary sites is the reservoir for *S. aureus* in non-lactating goats. Longitudinal sampling of young goats and genotype comparison between extramammary and IMI strains should provide insight into the role of extramammary site colonization, as a source for *S. aureus* IMI in pre-parturition primiparous goats. 

In conclusion, our results show that in goats, different extramammary sites (i.e., the nares, groin, vulva, hock and udder) can be colonized by mastitis-associated *S. aureus spa* genotypes. The *spa* genotypes t544 and t1236 were the dominant types in this herd and were isolated in equal proportions from the extramammary sites and milk. Extramammary sites could be reservoirs for *S. aureus* that are not targeted by current mastitis control measures and may also be reservoirs for IMI in pre-parturition primiparous goats.

## Figures and Tables

**Table 1:** Prevalence estimates for the colonization of extramammary sites and intramammary infections (milk) with *Staphylococcus aureus* in a Dutch dairy goat herd.

	Milk	Nares	Vulva	Hock	Groin	Udder Cleft	Extramammary Colonization ^1^
*Staphylococcus aureus* positive culture	15	54	6	8	3	7	62
Total	207	120	120	120	120	120	120
Observed prevalence (95% CI ^2^)	7.2% ^3^ (4.4–11.6)	45% (36.4–53.9)	5% (2.3–10.5)	6.7% (3.4–12.6)	2.5% (0.9–7.1)	5.8% (2.9–11.6)	51.7% (42.8–60.4)

^1^ The prevalence of goats colonized in at least one extramammary site, so excluding milk. ^2^ 95% confidence interval, Wilson method. ^3^ Period prevalence.

**Table 2:** *Staphylococcus aureus spa* genotypes identified in milk and in extramammary sites in a Dutch dairy goat herd. There was no association between the *spa* genotype and the site (milk or extramammary site) (*p* = 0.141, Fisher–Freeman–Halton exact test).

*Spa* Type	Location						
	Milk (%)	Extramammary Site (%)	Nares	Vulva	Hock	Groin	Udder Cleft
t426	1 (6.7%)	2 (3.2%)	1	1			
t544	8 (53.3%)	51 (82.3%)	42	4	6	2	6
t1236	5 (33.3%)	14 (22.6%)	12	1	1	1	1
t3992		1 (1.6%)	1				
Non-typeable ^1^		1 (1.6%)			1		
Novel type 1	1 (6.7%)						
Novel type 2		1 (1.6%)	1				
Total	15	62	57	6	8	3	7

^1^ Negative in the spa PCR.

## Data Availability

No application.

## Supplementary Materials

The following supporting information can be downloaded at: https://www.mdpi.com/article/10.3390/pathogens12040515/s1, Table S1: Overview of milk and extramammary body sites colonized with S. aureus and the spa genotypes identified from these sites in a Dutch dairy goat herd.
