# Supplementary material for: Colonization of Extramammary Sites with Mastitis-Associated S. aureus Strains in Dairy Goats
Source: Pathogens. 2023 Mar 26;12(4):515. doi: 10.3390/pathogens12040515 (PMC10140999; doi:10.3390/pathogens12040515)
Supplement: Supplementary file 1 [file pathogens-12-00515-s001.zip › pathogens-2286469-supplementary.pdf]

Supplementary Table S1: Overview of milk and extramammary body sites colonized with *S. aureus* and the *spa* genotypes identified from these sites in a Dutch dairy goat herd.

Animal ID	Milk	Nares	Vulva	Hock	Groin	Udder cleft
1	-[1]	t544	-	-	-	-
2	-	t544	-	-	-	-
3	-	t544/t1236	-	-	-	-
5	-	t544	-	-	-	-
6	-	t544	-	-	-	-
8	-	t1236	-	-	-	-
9	-	t544	-	t1236	-	t1236
10	-	t1236	-	-	-	-
11	-	t544	t544	-	-	-
12	-	t544	-	-	-	-
14	-	t544	-	-	-	-
15	-	t544	-	-	-	-
16	-	-	t1236	-	-	-
17	-	t544	-	-	-	-
18	-	t544	-	-	-	-
19	-	t544	-	-	-	-
21	-	t544	-	-	-	-
25	-	-	t426	Non-typeable	-	-
26	-	t1236	-	-	t1236	-
27	-	t1236	-	-	-	-
28	-	t426	-	-	-	-
31	-	t1236	-	-	-	t544
32	-	t544	-	-	-	-
33	-	-	t544	-	-	-
34	-	t544	-	-	-	t544
37	-	t544	-	-	-	-
39	-	t544	-	-	-	-
41	-	t544	-	-	t544	-
43	-	t544	-	-	-	-
44	-	t544	-	-	-	-
45	-	t544	-	t544	-	-
47	-	t544	-	-	-	-
49	-	t1236	-	-	-	-
50	-	t544	-	t544	-	-
51	t544	t544	-	-	-	-
53	-	-	-	t544	-	-
55	-	t1236	-	-	-	-
56	-	-	-	t544	-	-
59	-	t544	-	-	-	-
60	t544	t544	-	t544	-	-
68	-	t544	-	-	-	-
69	-	t544	-	-	-	-
70	-	-	t544	-	-	-
71	-	-	-	-	-	t544

74	-	t544/novel type 2	-	-	-	-
77	-	t544	-	-	-	-
79	-	t1236	-	-	-	-
81	-	t1236	t544	t544	-	-
84	-	-	-	-	-	t544
85	-	t544	-	-	-	-
86	-	t544	-	-	-	-
88	-	t544	-	-	-	-
89	-	t544	-	-	-	-
90	-	t544	-	-	-	t544
100	-	t544	-	-	-	-
101	-	t1236	-	-	t544	t544
103	-	t544	-	-	-	-
104	-	t544	-	-	-	-
112	-	t1236	-	-	-	-
114	-	t544	-	-	-	-
117	-	t544/t3992	-	-	-	-
120	t544	t544	-	-	-	-
ET33236[2]	t544					
ET33249	Novel type 1					
ET33423	t544					
ET33113	t1236					
ET44423	t1236					
ET33274	t544					
ET44153	t1236					
ET33194	t544					
ET33111	t426					
ET55065	t1236					
ET44068	t544					
ET33029	t1236					

[1] Culture negative for *S. aureus*.
[2] Animal ID numbers starting with ET are goats from which only milk samples were collected.
